# Ethanol induction of FGF21 in the liver is dependent on histone acetylation and ligand activation of ChREBP by glycerol-3-phosphate

**DOI:** 10.1073/pnas.2505263122

**Published:** 2025-05-29

**Authors:** Mi Cheong Cheong, Bryan Mackowiak, Hyung Bum Kim, Genaro Hernandez, Tulip Nandu, Kevin Vale, Yuan Zhang, Lauren G. Zacharias, Thomas P. Mathews, Bin Gao, W. Lee Kraus, Steven A. Kliewer, David J. Mangelsdorf

**Affiliations:** ^a^Department of Pharmacology, University of Texas Southwestern Medical Center, Dallas, TX 75390; ^b^Laboratory of Liver Diseases, National Institute on Alcohol Abuse and Alcoholism, NIH, Bethesda, MD 20892; ^c^Laboratory of Signaling and Gene Regulation, Cecil H. and Ida Green Center for Reproductive Biology Sciences, University of Texas Southwestern Medical Center, Dallas, TX 75390; ^d^Children’s Medical Center Research Institute, UT Southwestern Medical Center, Dallas, TX 75390; ^e^Department of Molecular Biology, UT Southwestern Medical Center, Dallas, TX 75390; ^f^HHMI, UT Southwestern Medical Center, Dallas, TX 75390

**Keywords:** ChREBP, FGF21, alcohol, liver, transcription

## Abstract

Ethanol rapidly stimulates the liver to synthesize the hormone fibroblast growth factor 21 (FGF21), which then acts on the brain to elicit a multifaceted protective response. We show that in mice, this induction of FGF21 occurs at the level of gene transcription and is regulated by two byproducts of ethanol metabolism, glycerol-3-phosphate (G3P) and acetyl-CoA. Using cell-based reporter and thermal shift binding assays, we show that G3P binds to a conserved domain and activates the transcription factor carbohydrate-responsive element-binding protein (ChREBP), which regulates the *Fgf21* gene promoter. The stimulation of *Fgf21* gene transcription by ethanol also requires its metabolism to acetyl-CoA and correlates with histone acetylation. Accordingly, a p300/CBP histone acetyltransferase inhibitor blocks histone acetylation, ChREBP recruitment, and transcriptional activation at the *Fgf21* promoter. Together, these findings reveal a dual regulatory mechanism driven by both G3P and acetyl-CoA that explains ethanol’s robust stimulatory effect on *Fgf21* and possibly other ChREBP target genes in the liver.

## Significance

Alcohol triggers the liver to produce the hormone FGF21, which helps protect against its toxic effects. However, how alcohol induces FGF21 synthesis is incompletely understood. We now show that two byproducts of alcohol metabolism, glycerol-3-phosphate (G3P) and acetyl-CoA, work together to stimulate transcription of the *Fgf21* gene in mice. G3P binds directly to the transcription factor ChREBP to activate it, and acetyl-CoA is used to modify histones at the *Fgf21* gene promoter, increasing its accessibility to ChREBP. This dual mechanism strongly induces the transcription of *Fgf21* and probably many other genes that are turned on in the liver by alcohol.

Animals that consume diets rich in fruits or other sources of simple carbohydrates have evolved mechanisms to neutralize and detoxify any accompanying ethanol produced by the natural fermentation process (1, 2). These adaptations include specialized enzymes, such as alcohol and aldehyde dehydrogenases, to metabolize ethanol efficiently. Ethanol also elicits adaptive changes in gene expression that protect against its toxic effects. For example, in the liver, ethanol induces the transcription of genes involved in catabolizing and converting ethanol to lipids and defending against inflammation and oxidative stress. The mechanisms underlying these ethanol-induced changes in gene transcription include activation of specific transcription factors and epigenetic modifications of histones and other regulatory proteins (3).

FGF21 is a hormone that is rapidly and robustly induced by ethanol in both human and murine liver (4–7). In mice, circulating FGF21 acts on its receptor complex in the brain to elicit a multifaceted protective response. This includes activating the noradrenergic nervous system to stimulate alertness as a means to counteract ethanol-induced intoxication (8); acting on the hypothalamus to stimulate water drinking to defend against dehydration (7); and activating an amygdalo-striatal circuit to suppress additional ethanol consumption (9–11). FGF21 also protects against liver inflammation and injury caused by chronic ethanol exposure (4, 12). These combined actions suggest that FGF21 could be exploited therapeutically to treat various alcohol use disorders.

In mice, induction of hepatic FGF21 by ethanol requires carbohydrate response element binding protein (ChREBP), a member of the Mondo family of bHLHZip transcription factors that is also activated by simple carbohydrates such as glucose and fructose (13–16). ChREBP binds as a heterodimer with Max-like protein (MLX) to carbohydrate response elements (ChoREs) located in the regulatory regions of FGF21 and other target genes (17–19). Ethanol is proposed to activate ChREBP through two distinct mechanisms. First, a study using hepatoma cells showed that ethanol-induced acetylation of ChREBP is required for its activation (20). Second, ethanol-induced reductive stress stimulates the synthesis of the triose phosphates glycerol-3-phosphate (G3P) and glyceraldehyde-3-phosphate (Ga3P), which have been proposed as possible allosteric activators of ChREBP (13).

In this report, we examine how ethanol induces FGF21 expression in murine liver. We show that ethanol stimulates *Fgf21* gene transcription through a two-pronged mechanism involving both direct binding of G3P to ChREBP and the acetylation of histones on the *Fgf21* gene promoter.

## Results

### Transcription of the *Fgf21* Gene Is Rapidly Induced By Ethanol Consumption.

**Fig. 1.**
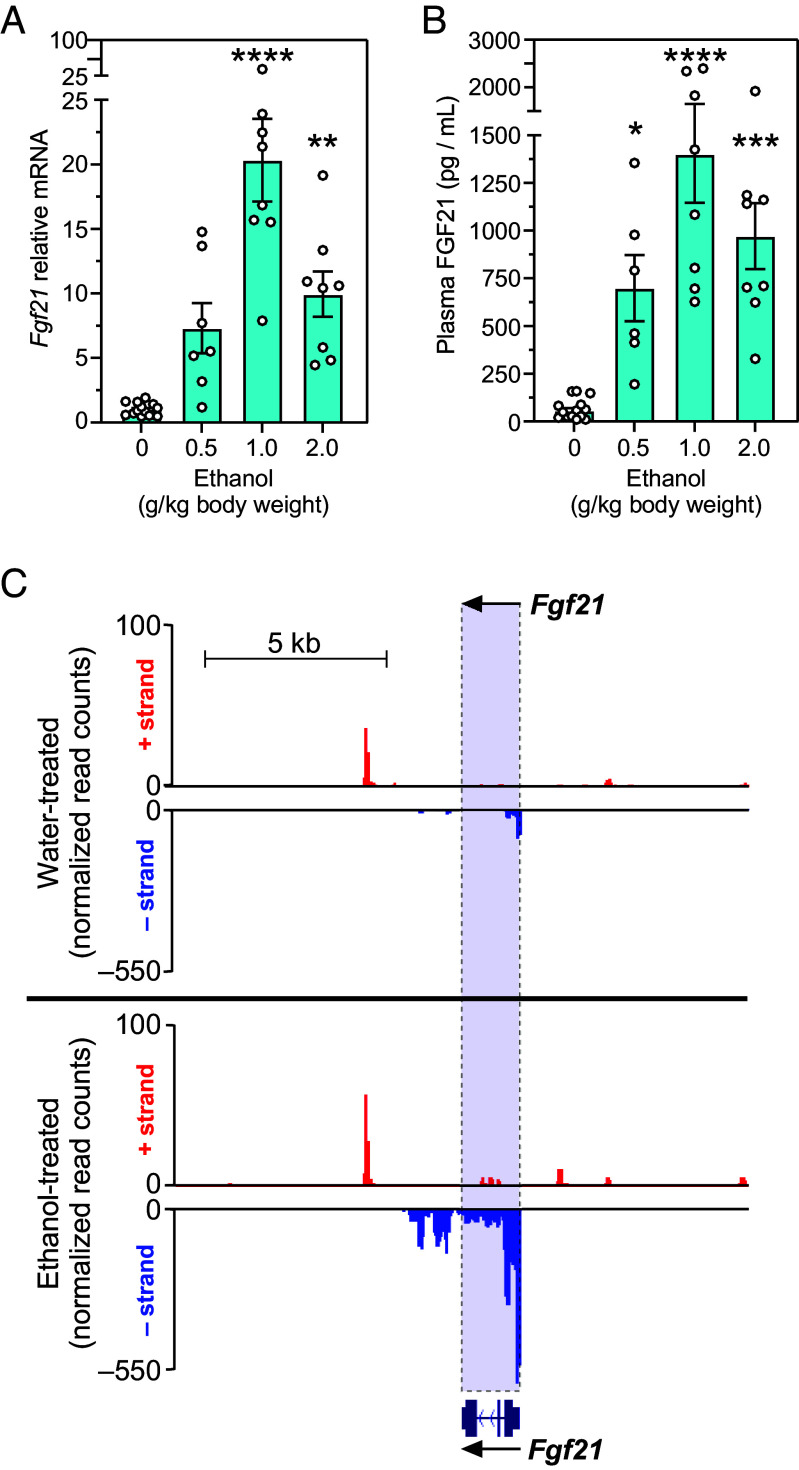
Ethanol stimulates hepatic FGF21 synthesis. (*A*) Hepatic *Fgf21* mRNA and (*B*) plasma FGF21 levels in C57BL/6 mice 1 h after giving a single bolus of water or increasing concentrations of ethanol by gavage. Values are means ± SEM. *P < 0.05, **P < 0.01, ***P < 0.001, ****P < 0.0001 for water versus ethanol gavage groups (n = 6 to 8) by one-way ANOVA with Tukey’s multiple comparisons test. (*C*) Genome browser tracks of PRO-seq data for the *Fgf21* gene in the livers of C57BL/6 mice 1 h after giving water or ethanol (1 g/kg) by oral gavage. Browser tracks show merged data from 3 mice per group. Note that the *Fgf21* gene is transcribed from the bottom (–) strand.

To identify optimal conditions for FGF21 induction by ethanol, we performed a dose–response analysis. Mice were administered ethanol at doses ranging from 0.5 to 2 g/kg by oral gavage, and liver and blood were collected 1 h later. Induction of both *Fgf21* mRNA in the liver and FGF21 protein in plasma was maximal at the 1 g/kg dose (Fig. 1 *A* and *B*). Complementary precision run-on sequencing (PRO-seq) analysis demonstrated that this rapid, 1-h induction of FGF21 occurred at the level of gene transcription (Fig. 1*C* and *SI Appendix*, Dataset S1).

### Metabolism of Ethanol to Acetate Is Required for *Fgf21* Induction.

**Fig. 2.**
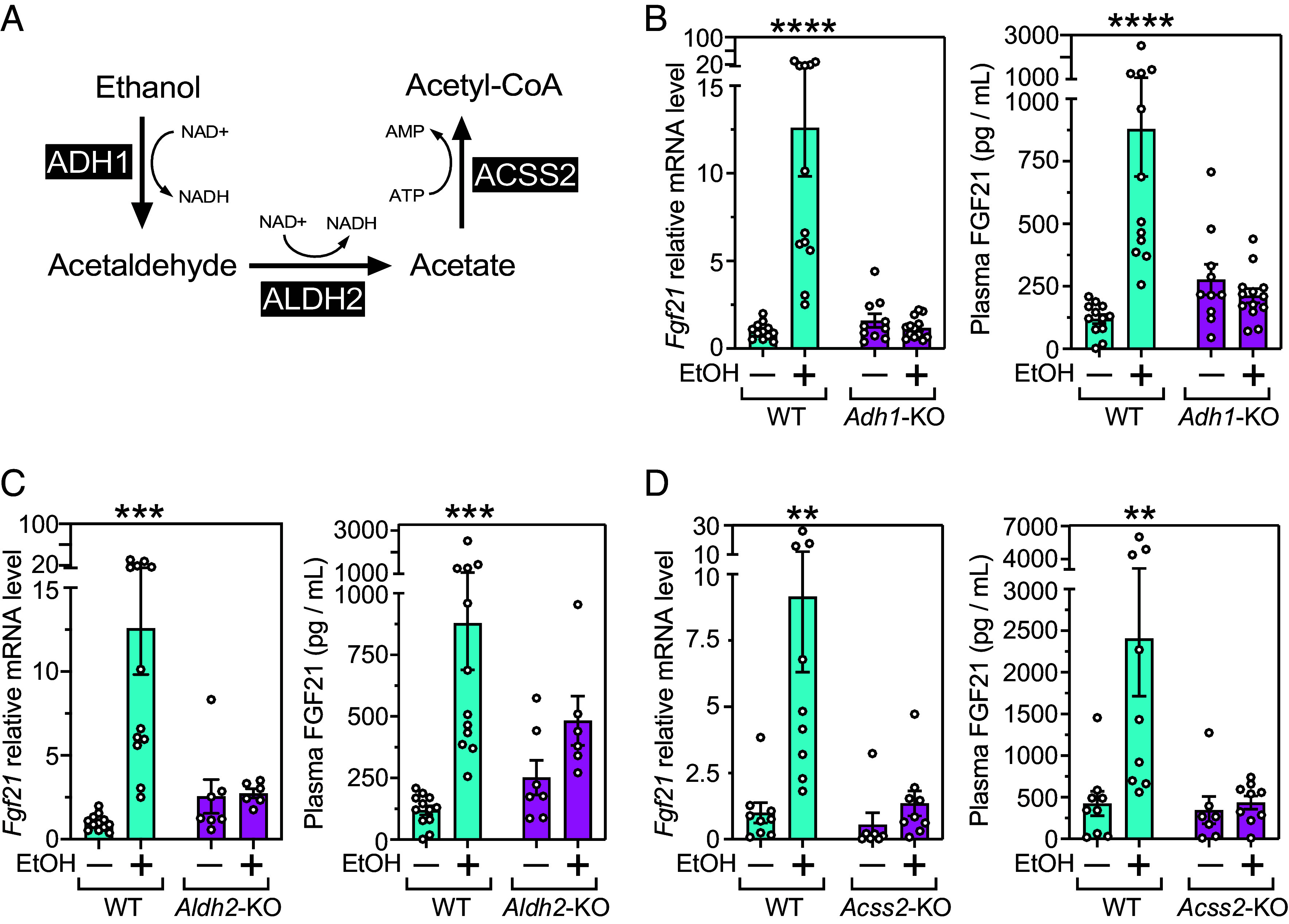
Ethanol induction of FGF21 synthesis requires metabolism to acetyl-CoA. (*A*) Schematic showing ethanol metabolism by alcohol dehydrogenase 1 (ADH1), aldehyde dehydrogenase 2 (ALDH2), and acyl-CoA synthetase short-chain family member 2 (ACSS2). (*B*–*D*) Hepatic *Fgf21* mRNA and plasma FGF21 levels in global *Adh1*-KO (n = 10 to 13), *Aldh2*-KO (n = 6 to 12), or *Acss2*-KO (n = 7 to 12) mice and their corresponding wild-type (WT) (n = 12) mice 1 h after giving water (–) or 1 g/kg ethanol (EtOH) (+) by oral gavage. Values are means ± SEM. **P < 0.01, ***P < 0.001, ****P < 0.0001 for water versus ethanol gavage groups by two-way ANOVA with Tukey’s multiple comparisons test.

To determine whether ethanol catabolism is required for FGF21 induction in vivo, we measured ethanol-mediated induction of FGF21 liver mRNA and plasma protein concentrations in mice lacking either alcohol dehydrogenase 1 (ADH1) or aldehyde dehydrogenase 2 (ALDH2), which are the principal ethanol metabolizing dehydrogenases in murine liver (Fig. 2*A*). Ethanol induction of FGF21 was eliminated in both these knockout (KO) models (Fig. 2 *B* and *C*). Likewise, FGF21 induction was eliminated in mice lacking acetyl-CoA synthetase 2 (ACSS2), which converts acetate into acetyl-CoA (Fig. 2 *A* and *D*). These data demonstrate that catabolism of ethanol to acetyl-CoA is required for efficient induction of FGF21.

### Direct Acetylation of ChREBP Is not Required for its Activation By Ethanol.

**Fig. 3.**
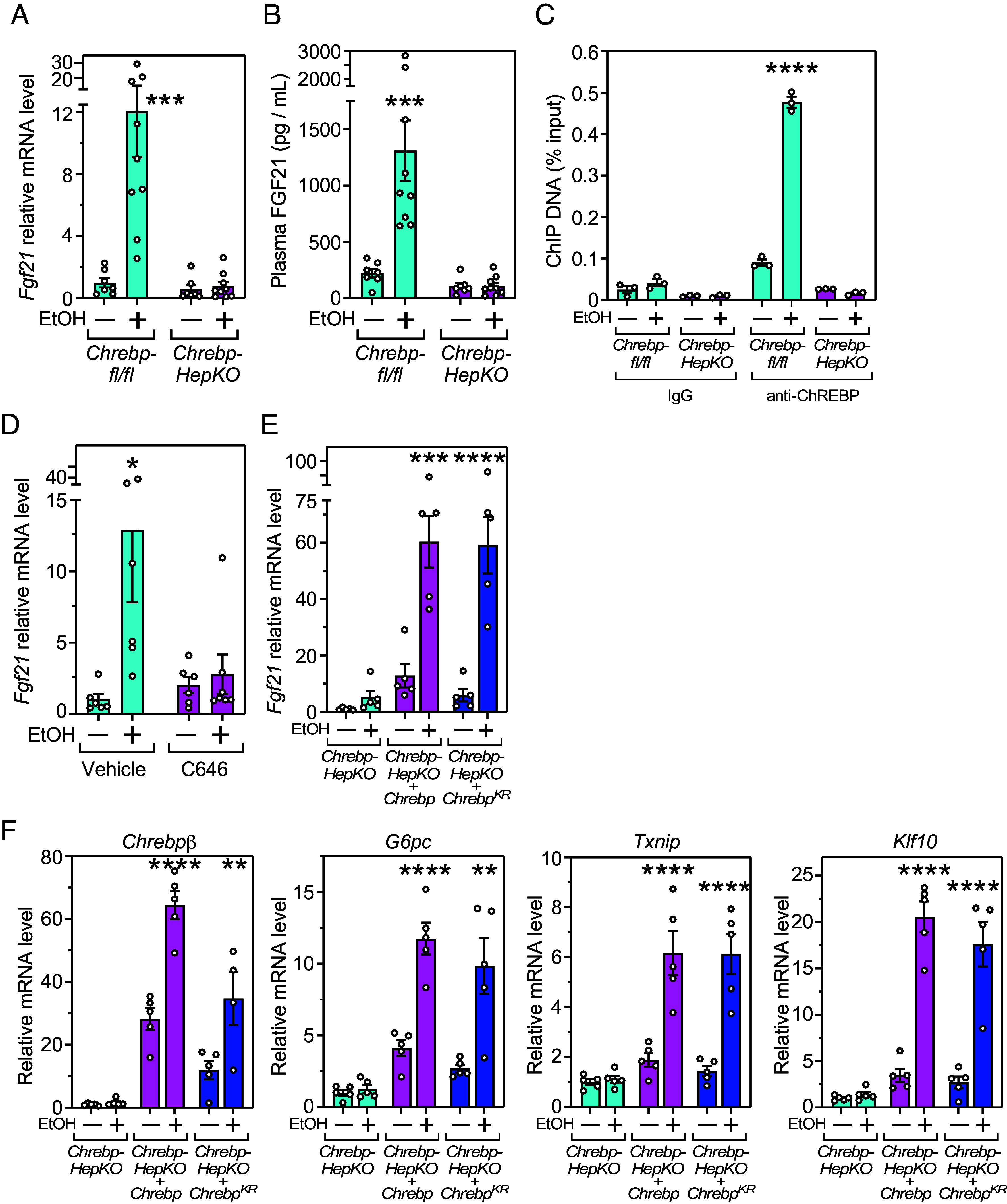
ChREBP-dependent induction of *Fgf21* and other genes by ethanol does not require ChREBP acetylation. (*A*) Hepatic *Fgf21* mRNA and (*B*) plasma FGF21 levels in control *Chrebp^fl/fl^* (n = 7 to 9) and *Chrebp-HepKO* (n = 7 to 9) mice 1 h after giving water (–) or 1 g/kg ethanol (+) by oral gavage. (*C*) ChIP analysis showing the effect of ethanol on ChREBP binding to the *Fgf21* promoter in mouse livers (n = 3). (*D*) Hepatic *Fgf21* mRNA in WT mice 1 h after giving either water (–) or 1 g/kg ethanol (+) by oral gavage plus vehicle or p300/CBP inhibitor (C646, 20 mg/kg) by i.p. injection n = 6 to 7). (*E*) Hepatic *Fgf21* mRNA levels in *Chrebp-HepKO*, *Chrebp-HepKO* + *Chrebp,* and *Chrebp-HepKO* + *Chrebp^KR^* mice 1 h after giving water (–) or 1 g/kg ethanol (+) by oral gavage (n = 5). (*F*) Hepatic mRNA levels for *Chrebpβ*, glucose-6-phosphatase (*G6pc*), thioredoxin-interacting protein (*Txnip*), and Krüppel-like factor 10 (*Klf10*) in *Chrebp-HepKO*, *Chrebp-HepKO* + *Chrebp,* and *Chrebp-HepKO* + *Chrebp^KR^* mice under the same conditions as in (*E*) (n = 4 to 5). To create hepatocyte knockouts of ChREBP, *Chrebp^fl/fl^* mice were infected with hepatocyte-selective AAVs expressing GFP (*Chrebp^fl/fl^*) or CRE (*Chrebp*-*HepKO*). To test the effects of acetylation, *Chrebp^fl/fl^* mice were infected with an AAV expressing CRE together with AAVs expressing wild-type ChREBP (*Chrebp-HepKO*+*Chrebp*) or the acetylation sites mutant ChREBP (*Chrebp*-*HepKO+Chrebp^KR^*). Values are means ± SEM. *P < 0.05, **P < 0.01, ***P < 0.001 and ****P < 0.0001 for water versus ethanol gavage groups by two-way ANOVA with Tukey’s multiple comparisons test.

Induction of hepatic FGF21 by ethanol was previously shown to be eliminated in global *Chrebp*-KO mice (13). We observed this same result in mice in which ChREBP was selectively eliminated in hepatocytes (*Chrebp*-HepKO) (Fig. 3 *A* and *B*). Likewise, ethanol induction of other ChREBP target genes, including *Chrebpβ,* which encodes a shorter ChREBP isoform derived from a distinct promoter and alternative first exon, glucose-6-phosphatase (*G6pc*), thioredoxin-interacting protein (*Txnip*), and Krüppel-like factor 10 (*Klf10*), was lost in *Chrebp*-HepKO mice (*SI Appendix*, Fig. S1*A*). Ethanol exposure did not affect mRNA levels of *Mlxγ*, which encodes ChREBP’s DNA binding partner (*SI Appendix*, Fig. S1*A*). In chromatin immunoprecipitation studies performed with liver, ChREBP was efficiently recruited to the *Fgf21* promoter in response to ethanol administration in control but not *Chrebp*-HepKO mice (Fig. 3*C*).

ChREBP can be bound and activated by the histone acetyltransferase, p300, which acetylates ChREBP at residues K658, K672, and K678 in its DNA binding domain (21). A previous study showed that ethanol induced the acetylation of ChREBP in murine and human hepatocytes and that an acetylation site mutant (K672A) blunted ethanol-induced activation of ChREBP in a cell-based reporter assay (20). Consistent with the possibility that ethanol activates ChREBP via p300-mediated acetylation, treatment of mice with the p300/CBP inhibitor, C646, significantly inhibited ethanol induction of hepatic *Fgf21*, *Chrebpβ* and *G6pc*, while also causing *Txnip* and *Klf10* induction to trend lower (Fig. 3*D* and *SI Appendix*, Fig. S1*B*).

To test directly whether ethanol regulates FGF21 by inducing the acetylation of ChREBP, we introduced a ChREBP mutant (ChREBP^KR^) lacking all three previously characterized acetylation sites into livers of mice in which endogenous ChREBP expression had been knocked out. Mice expressing either this acetylation site mutant or wild-type (WT) ChREBP, which had been reintroduced at comparable concentrations (*SI Appendix*, Fig. S2*A*), were administered ethanol, and *Fgf21* expression was measured in the liver. Surprisingly, *Fgf21* induction was completely intact in mice expressing the acetylation sites mutant (Fig. 3*E*). *Chrebpβ*, *G6pc*, *Txnip*, and *Klf10* were also still induced by ethanol (Fig. 3*F*). We conclude that while ChREBP is required for ethanol to induce each of these genes, its direct acetylation at these three lysines is not. However, this does not rule out the possibility of effects from acetylation at other sites.

### Ethanol Induces Histone H3K9 Acetylation at the *Fgf21* Promoter.

**Fig. 4.**
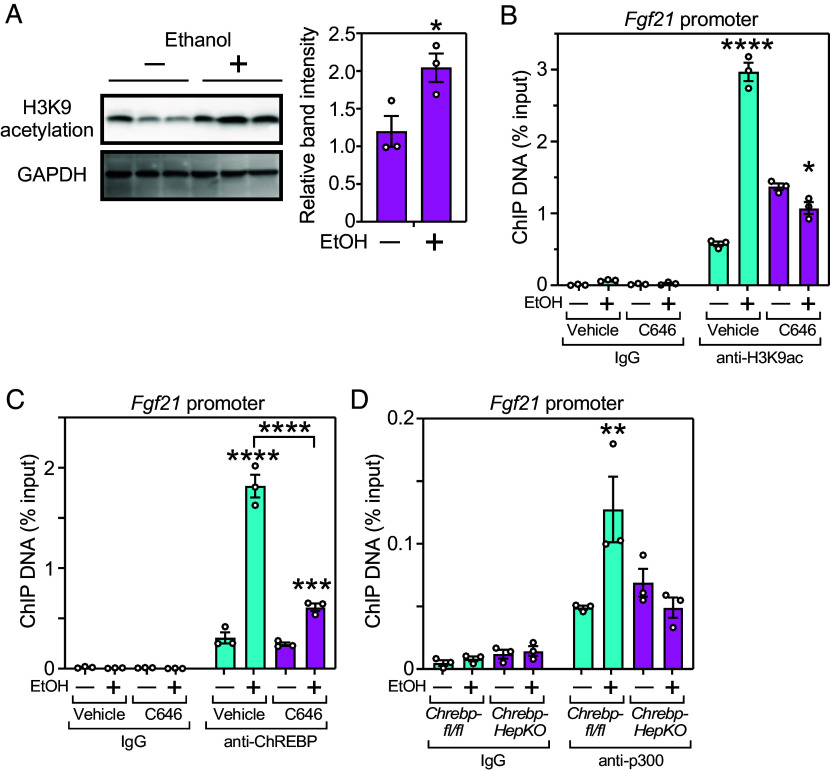
Ethanol-mediated induction of FGF21 requires histone acetylation of the *Fgf21* promoter. (*A*) Western blot images (*Left* panel) and quantification (*Right* panel) of H3K9 acetylation of the *Fgf21* promoter 1 h after giving water (–) or 1 g/kg ethanol (+) by oral gavage to WT mice. Values are means ± SEM. *P < 0.05 by unpaired *t* test. (*B*) ChIP analysis showing H3K9 acetylation of the *Fgf21* promoter in mouse liver 1 h after giving water (–) or 1 g/kg ethanol (+) by oral gavage plus vehicle or p300/CBP inhibitor (C646, 20 mg/kg) by i.p. injection. Immunoprecipitation was performed with IgG or anti-H3K9ac antibodies. (*C*) ChIP analysis of ChREBP binding to the hepatic *Fgf21* promoter under the same conditions as in (*B*). (*D*) ChIP analysis demonstrating p300 binding to the hepatic *Fgf21* promoter 1 h after giving water (–) or 1 g/kg ethanol (+) by oral gavage to control *Chrebp^fl/fl^* or *Chrebp-HepKO* mice (n = 3). Data are expressed as means ± SEM from n = 3 individual mice per group. In (*B*–*D*) *P < 0.05, **P < 0.01, and ****P < 0.0001 by two-way ANOVA with Tukey’s multiple comparisons test comparing water to EtOH treatment in each group unless otherwise noted.

To explore other possible mechanisms whereby p300/CBP-mediated acetylation stimulates *Fgf21* expression, we analyzed histones at the *Fgf21* promoter. Since it was previously reported that ethanol stimulates H3K9 acetylation in murine liver (22, 23), we focused on this epigenetic modification. Ethanol administration to mice increased acetylated H3K9 concentrations in both the whole liver and at the *Fgf21* gene promoter (Fig. 4 *A* and *B*). Treatment of mice with the p300/CBP inhibitor, C646, increased basal H3K9 acetylation at the *Fgf21* promoter possibly by increasing the expression level of other histone acetyltransferases as previously described (24). However, C646 inhibited any further induction and ChREBP recruitment by ethanol (Fig. 4 *B* and *C*). Accordingly, ethanol stimulated p300 recruitment to the *Fgf21* promoter through a ChREBP-dependent mechanism (Fig. 4*D*). These data suggest that the acetyl-CoA derived from ethanol induces *Fgf21* expression by stimulating p300-mediated histone acetylation at its promoter. The C646 data discussed above further suggest that this mechanism extends to other ChREBP target genes (*SI Appendix*, Fig. S1*B*).

### G3P Binds and Activates ChREBP.

**Fig. 5.**
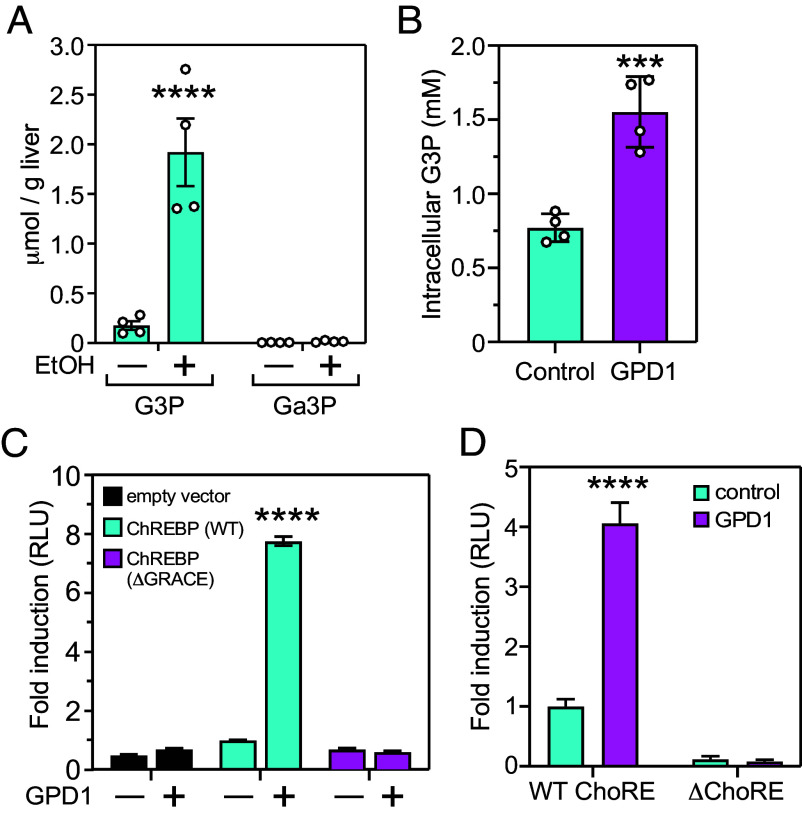
Glycerol-3-phosphate (G3P) activates ChREBP. (*A*) G3P and glyceraldehyde-3-phosphate (Ga3P) concentrations in mouse liver 1 h after giving water (–) or 1 g/kg ethanol (+) by oral gavage. (*B*) G3P concentrations in HEK-293T cells in either the absence or presence of GPD1 coexpression. (*C*) The GRACE domain of ChREBP is required for G3P induction of the *Fgf21* promoter. Luciferase reporter assay of the *Fgf21* promoter in HEK-293T cells expressing either WT ChREBP or ChREBP–ΔGRACE in the absence (–) or presence (+) of GPD1 coexpression. (*D*) Deletion of the ChREBP response element (ΔChoRE) in the *Fgf21* promoter abolishes G3P-mediated transcription. In (*C* and *D*) HEK-293T cells were cotransfected with empty vector or vectors expressing WT ChREBP, GRACE-deleted (ΔGRACE) ChREBP, or GPD1, as indicated, and either a WT *Fgf21*-luciferase reporter plasmid (in panel *C* and *D*) or an *Fgf21*-luciferase reporter plasmid lacking the ChoRE (ΔChoRE, in panel *D*). All values are means ± SEM. ***P < 0.001, ****P < 0.0001. In (*A*, *C*, and *E*), p-values refer to comparisons versus no ethanol or GPD1 by two-way ANOVA with Tukey’s multiple comparisons test. Significance in (*B*) was determined by an unpaired *t* test.

ChREBP is activated by reductive stress through a mechanism that correlates with increased intracellular concentrations of the triosephosphates G3P and Ga3P (13). To examine the potential contribution of G3P and Ga3P to induction of FGF21, we measured their concentrations in the livers of mice administered ethanol. While G3P was increased ~10-fold to millimolar concentrations, Ga3P concentrations were much lower and not significantly increased by ethanol (Fig. 5*A*).

To test whether G3P activates ChREBP, we performed cell-based reporter assays using HEK-293T cells, which express little or no ChREBP (*SI Appendix*, Fig. S2*B*). Cells were transfected with an *Fgf21* promoter-luciferase reporter plasmid containing a ChoRE together with expression plasmids for G3P dehydrogenase (GPD1), which synthesizes G3P from dihydroxyacetone phosphate, MLX, which is ChREBP’s binding partner, and either WT ChREBP or ChREBP-ΔGRACE, which lacks the domain that has been proposed as a binding site for allosteric activators (25). Expression of GPD1, WT ChREBP, and ChREBP-ΔGRACE was confirmed by immunoblot analysis (*SI Appendix*, Fig. S2*B*). GPD1 expression increased intracellular G3P concentrations as expected (Fig. 5*B*) but did not affect reporter activity in the absence of ChREBP (Fig. 5*C*). However, coexpression of GPD1 with WT ChREBP, but not ChREBP-ΔGRACE, strongly induced reporter activity (Fig. 5*C*). GPD1’s effect in the reporter assay was abolished by deleting the ChoRE in the *Fgf21* promoter-luciferase reporter plasmid (Fig. 5*D*). These data suggest that G3P activates ChREBP through a GRACE-dependent mechanism.

**Fig. 6.**
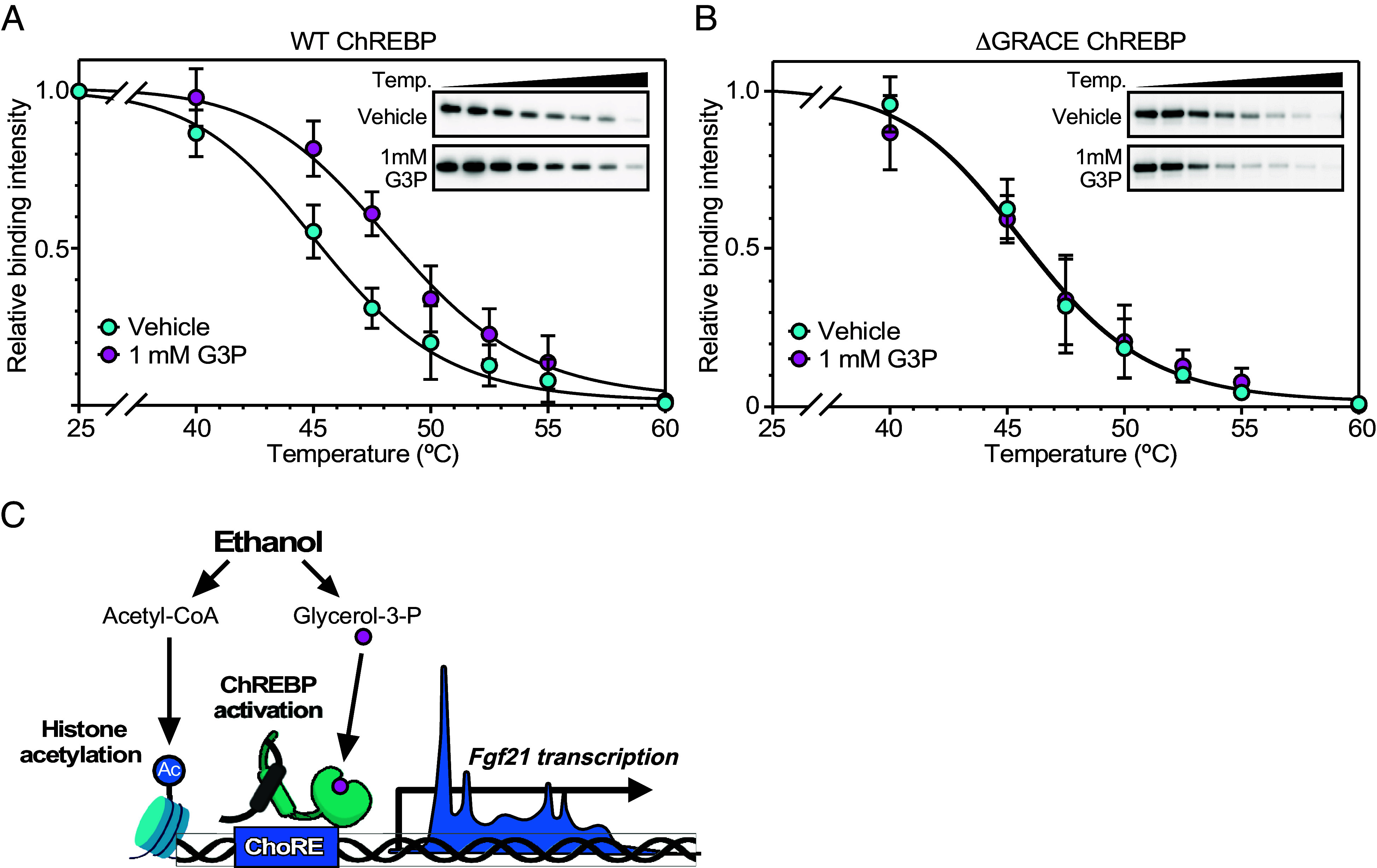
G3P binds to ChREBP. (*A* and *B*) Thermal shift analysis was performed using lysates from HEK-293T cells expressing WT or GRACE-domain deleted (ΔGRACE) ChREBP that were treated with vehicle or 1 mM G3P for 30 min. Insets show representative thermal shift stabilities of ChREBP proteins. (*C*) Model of the dual mechanism for activation of *Fgf21* gene expression by ethanol.

We used a thermal shift assay, which is based on the biophysical principle of ligand-induced thermal stabilization of target proteins (26), to examine whether G3P binds directly to ChREBP. Extracts from HEK-293T cells expressing ChREBP were incubated with either G3P or vehicle followed by heating at different temperatures to induce protein unfolding. Aggregated (unliganded) ChREBP was removed by centrifugation and the remaining soluble (liganded) ChREBP quantified by western blot analysis. Importantly, G3P increased the thermal stability of ChREBP, indicating a direct interaction between G3P and ChREBP (Fig. 6*A*). G3P had no effect on the thermal stability of a ChREBP derivative lacking the GRACE domain (Fig. 6*B*). We conclude that G3P binds directly to ChREBP in a GRACE-dependent manner. Another group also recently showed that glycerol-3-phosphate binds to the ChREBP GRACE domain (27).

## Discussion

While hepatic FGF21 expression is strongly induced by diverse metabolic stresses in mice, the response in humans is consistently much weaker. However, FGF21’s robust induction by ethanol is conserved between these species (4–7). In this report, we demonstrate by PRO-seq analysis that the rapid induction of *Fgf21* by ethanol occurs at the level of gene transcription. A recent study showed that ethanol’s stimulatory effect on *Fgf21* required ChREBP and correlated with triose phosphate concentrations, which are induced by reductive stress (13). We now provide several lines of evidence that one of these triose phosphates, G3P, serves as a ChREBP ligand. First, G3P activates ChREBP in cell-based reporter assays. Second, G3P binds directly to ChREBP as measured in thermal shift assays. Third, both G3P binding and activation require ChREBP’s GRACE domain, which has been proposed to serve as a binding site for allosteric activators (25). Finally, binding and activation occur at G3P concentrations consistent with those present in the liver following ethanol exposure. Together, these data provide strong evidence that ChREBP is an intracellular receptor for G3P.

We further demonstrate that efficacious induction of *Fgf21* also requires ethanol to be metabolized to acetate and converted to acetyl-CoA. A previous study showed that ethanol stimulated the acetylation of ChREBP in both human and murine hepatocytes and that an acetylation-defective mutant inhibited ethanol from activating ChREBP in a cell-based reporter assay (20). While these data suggested that ethanol activates ChREBP by inducing its acetylation, we show that a ChREBP mutant lacking all three established acetylation sites still induces *Fgf21* and other ChREBP target genes in the livers of mice administered ethanol. As an alternative mechanism, we show that ethanol stimulates p300 recruitment and H3K9 acetylation at the *Fgf21* promoter, and that ChREBP recruitment and *Fgf21* induction by ethanol are blocked by the p300/CBP histone acetyltransferase inhibitor C646. Our data suggest that the acetyl-CoA derived from ethanol metabolism stimulates ChREBP-mediated transcription by driving p300- and possibly CBP-mediated histone acetylation.

In closing, our findings reveal a dual mechanism for hepatic FGF21 induction by ethanol that involves both activation of ChREBP by G3P, which is driven by reductive stress, and the acetylation of histones at the *Fgf21* gene promoter, which requires acetyl-CoA (Fig. 6*F*). This model provides a mechanistic basis for ethanol’s rapid and efficacious stimulation of *Fgf21* gene transcription. Since ethanol induction of other genes is inhibited by both ChREBP knockout and C646, and metabolism of glucose and fructose also increases G3P and acetyl-CoA concentrations, this dual mechanism is likely to extend to other ChREBP target genes and activators.

## Materials and Methods

### Mouse Experiments.

All animal procedures were approved by UT Southwestern Medical Center’s Institutional Animal Care and Use Committee or the National Institute on Alcohol Abuse and Alcoholism’s Animal Care and Use Committee. Mice were housed in a temperature-controlled environment (23 ± 1 °C) with 12 h light/dark cycles and fed standard rodent chow ad libitum. Experiments were performed with male mice, which were randomly assigned to experimental groups. Global *Adh1*-KO (28) and *Aldh2*-KO (29) mice were backcrossed to C57BL6N background for more than 10 generations, and corresponding control C57BL6N mice were purchased from Charles River Laboratory. Global *Acss2*-KO (C57/BL6J background) (30) and *Chrebp^fl/fl^* mice (mixed C57BL6J/C57BL6N background) (31) were previously described. WT C57BL6J (Strain #000664, 8 wk old) were purchased from Jackson Laboratory.

Hepatocyte-specific *Chrebp*-KO mice were generated by injecting *Chrebp^fl/fl^* mice with AAV8-TBG-Cre or control AAV8-TBG-GFP (Addgene) at a dose of 1 × 10^11^ genome copies/mouse. Infected mice were maintained for two weeks before use in experiments. For the ChREBP acetylation sites mutant experiments, *Chrebp^fl/fl^* mice were injected with either AAV8-TBG-Cre + AAV8-TBG-GFP to generate control *Chrebp*-KO mice; AAV8-TBG-Cre + AAV8-TBG-*Chrebp* to generate WT ChREBP knockin mice; or AAV8-TBG-Cre + AAV8-TBG-*Chrebp^KR^* mutant to generate the triple lysine mutant (K658R/K672R/K678R) knockin mice. AAV plasmids were generated by Vectorbuilder and packaged by UT Southwestern’s Viral Vector Core.

For ethanol experiments, mice were administered ethanol (Pharmco-Aaper) diluted to 7% with water or water alone as a control by oral gavage using 20-gauge (1.5 inch) sterile gavage needles. Mice were killed 1 h later. For C646 treatment, mice were injected intraperitoneally with 20 mg/kg of C646 or vehicle. One hr later, mice were administered 1 g/kg of ethanol or water by oral gavage.

### Precision Run-On Sequencing (PRO-seq).

PRO-seq run-on, library preparation, and analysis were done as described (32) using approximately 5 million mouse liver nuclei per reaction. Libraries were prepared using three biological replicates per condition. The data for the *Fgf21* gene were extracted and are shown as browser tracks. The full dataset with methods and detailed analyses will be presented elsewhere.

### FGF21 ELISA.

Mice were killed by decapitation, and plasma was collected using EDTA (Sarstedt) following centrifugation. Plasma FGF21 levels were measured using ELISA kits (Biovendor and R&D Systems) according to the manufacturers’ protocols.

### QPCR Analysis.

RNA from murine liver was extracted using RNA Stat-60 (Iso-Tex Diagnostics). Complementary DNA (cDNA) was generated from RNA (4 μg) using High-Capacity cDNA Reverse Transcription Kits (Invitrogen). QPCR was performed by the SYBR Green method (33). Primer sequences for the genes analyzed are shown in *SI Appendix*, Table S1. Cyclophilin (NM_011149) was used as the reference mRNA.

### Chromatin Immunoprecipitation (ChIP) Assays.

ChIP assays were performed using SimpleChIP Plus Enzymatic Chromatin IP Kits with magnetic beads (Cell Signaling Technology). For ChREBP and acetylated H3K9, 25 mg of mouse liver was used for each immunoprecipitation, and 2% of the total isolated digested chromatin was used as input together with antibodies (Novus Biologicals NB400-135 for ChREBP; Abcam ab4441 for acetylated H3K9). For p300, 125 mg of mouse liver was used together with antibody (Active Motif 61401). Primer sequences for the *Fgf21* ChoRE were Fwd 5’ AGCCCTTTTCATTCAGACCCCT 3’ and Rev 5’ CTCCTGTGTTGAATCCCCAGCTGA 3’. Primer sequences for p300 were Fwd 5’ CTCAGACCCAAGAGCTAGATCC3’ and Rev 5’ AGGAGGCTGGGGTCTACACT3’.

### G3P and Ga3P Quantitation.

For analysis of liver concentrations, glycerol-3-phosphate (G3P) and glyceraldehyde-3-phosphate (Ga3P) were extracted from liver sections and analyzed by liquid chromatography-mass spectrometry (LC/MS) according to previously reported methods (34, 35). Alongside liver extracts, a calibration curve for G3P and Ga3P was prepared at 200 nM, 500 nM, 1 μM, 2 μM, 5 μM, 10 μM, 20 μM, and 50 μM. [^13^C_3_]-Ga3P and [^13^C_3_]-G3P internal controls were also added at a final concentration of 1 μM to each calibrator and liver extract. After acquisition, TraceFinder (ThermoScientific, San Jose, CA) was used to generate extracted ion chromatograms (XICs) for the theoretical masses of G3P (*m/z* 171.0064), Ga3P (*m/z* 168.9907), [^13^C_3_]-G3P (*m/z* 172.0008), and [^13^C_3_]-Ga3P (*m/z* 174.0165) in negative ionization mode with a 5 ppm mass tolerance. Each peak was reviewed for quality and integrated in all calibrators and extracted samples. Next, TraceFinder was used to plot the ratios of unlabeled metabolites to labeled metabolites for G3P and Ga3P in our calibration curve. These ratios were fit to a linear regression equation such that each calibration curve had an R^2^ value above 0.99, and the calculated accuracy of each calibrator was within 15% of their known concentrations. Finally, the ratios of native metabolites to isotopically labeled standards were calculated in each of the liver extracts and used together with the calibration curves to calculate the µM concentrations of G3P and Ga3P in extracts. G3P concentrations were measured in HEK-293T cell extracts using a kit (Abcam; ab174094) according to the manufacturer’s instructions.

### Cell-Based Reporter Assays.

pcDNA3.1-ChREBP, pcDNA3.1-Mlxγ, and pcDNA3.1-GDP1 expression plasmids were generated using mouse ChREBP (accession number NM_021455.5), Mlxγ (accession number NM_011550.3), and Gpd1 (accession number NM_005276.3). The ΔGRACE-ChREBP expression plasmid removed nucleotides encoding amino acids 197 to 298 from ChREBP. The mouse *Fgf21* promoter (−1497/+5)-luciferase reporter construct was described previously (36). The ΔChoRE mutant was generated using primers Fwd 5’ GCGGGCCTGTCTGGGTATAAA 3’ and Rev 5’ GCGTGATATTTGACACACTTGGCA 3’. For transfection experiments, HEK-293T cells were cultured in 10% fetal bovine serum in DMEM (Invitrogen) with antibiotics. Cells were transfected using Fugene HD (Promega) in 12-well plates. Transfections typically included 50 ng each of pcDNA3.1-ChREBP, pcDNA3.1-Mlxγ, pcDNA3.1-GPD1 plasmid, 5 ng of pRL-CMV (Renilla luciferase control), and 200 ng of either *Fgf21* promoter-luciferase or the ΔChoRE mutant per well. The empty vector control pcDNA3.1 was included when necessary to equalize the transfected plasmid amounts across wells. After 24 to 48 h, cells were washed once in 1× PBS, lysed in 1× passive lysis buffer (Promega), and incubated for 15 min on an orbital shaker before measuring luciferase activity with a dual-luciferase assay kit (Promega).

### Thermal Shift Assays.

G3P binding to ChREBP was measured in cell extracts by modifying a previously published thermal shift protocol (37). HEK-293T cells cultured in DMEM were transfected with 1 μg pcDNA3.1-ChREBP or pcDNA3.1-ΔGRACE-ChREBP using Lipofectamine 3000 (Invitrogen) in six-well plates. Cell lysates were prepared by three freeze–thaw cycles and incubated with 1 mM G3P or vehicle for 30 min followed by sequential heating in a PCR thermocycler at 40, 42.5 45, 47.5 50, 52.5, and 57.5 °C for 5 min. Extracts were centrifuged at 20,000×*g* for 10 min at 4 °C, and the soluble material was analyzed by western blot using an anti-ChREBP antibody. Relative band intensities were measured using Image J.

### Quantification and Statistical Analysis.

All data are expressed as means ± SEM. All reported sample sizes (*n*) represent a biologically independent experiment. Statistical analysis between two groups was performed by unpaired two-tailed Student’s *t* test using GraphPad Prism (GraphPad Software). For multiple comparisons, two-way ANOVA with post hoc Tukey was performed using GraphPad Prism.

## Supplementary Material

Appendix 01 (PDF)

Dataset S01 (XLSX)

## Data Availability

All study data are included in the article and/or supporting information.
