# Supplementary material for: Ethanol induction of FGF21 in the liver is dependent on histone acetylation and ligand activation of ChREBP by glycerol-3-phosphate
Source: Proc Natl Acad Sci U S A. 2025 May 29;122(22):e2505263122. doi: 10.1073/pnas.2505263122 (PMC12146743; doi:10.1073/pnas.2505263122)
Supplement: Supplementary file 1 — Appendix 01 (PDF) [file pnas.2505263122.sapp.pdf]

PNAS

Supporting Information for

Ethanol Induction of FGF21 in Liver is Dependent on Histone Acetylation and Ligand Activation of ChREBP by Glycerol-3-phosphate

Mi Cheong Cheong, Bryan Mackowiak, Hyung Bum Kim, Genaro Hernandez, Tulip Nandu, Kevin Vale, Yuan Zhang, Lauren G. Zacharias, Thomas P. Mathews, Bin Gao, W. Lee Kraus, Steven A. Kliewer, David J. Mangelsdorf

Email: steven.kliewer@utsouthwestern.edu, davo.mango@utsouthwestern.edu

This PDF file includes:

Figures S1 and S2
Table S1

Other supporting materials for this manuscript include the following:

Dataset S1

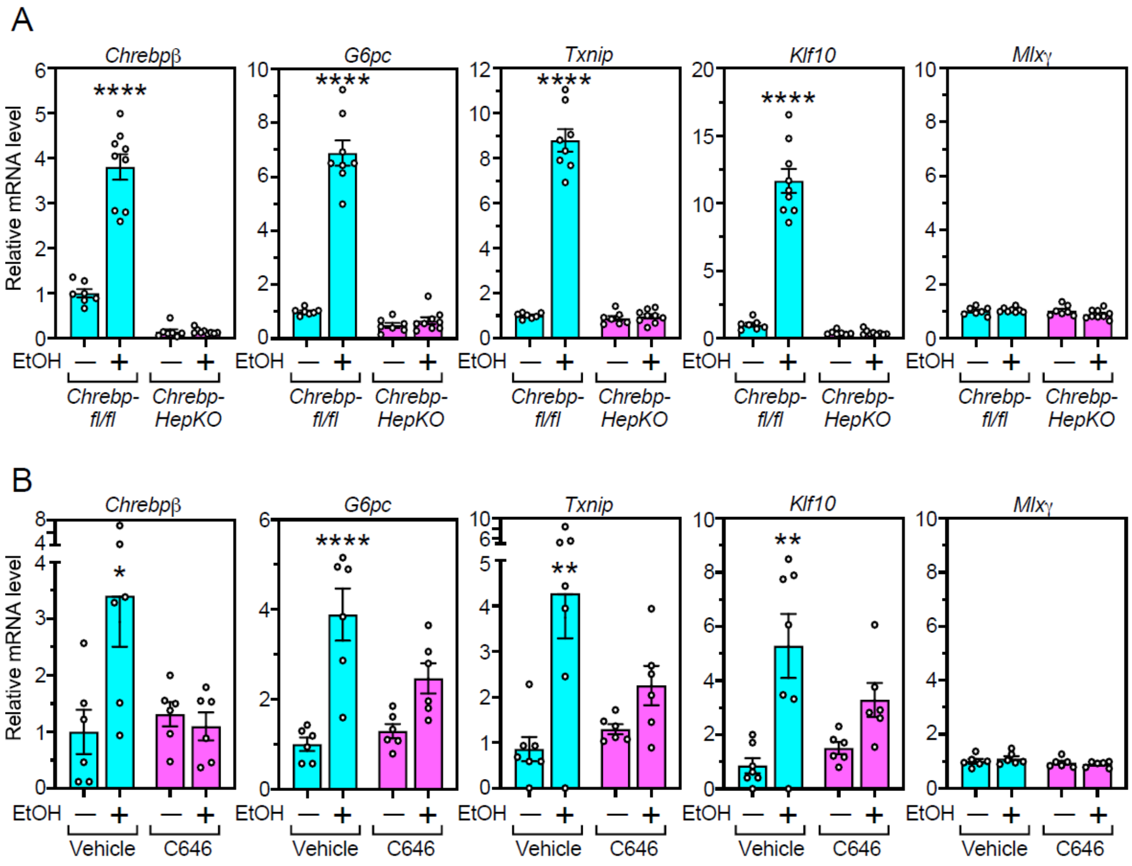

Fig. S1. Effects of ChREBP knockout or a p300/CBP inhibitor on ethanol induction of gene expression.

(A) Hepatic mRNA expression of the indicated genes in control *Chrebp*$^{fl/fl}$ (n=7-9) and *Chrebp-HepKO* (n=7-9) mice 1 h after giving water (–) or 1 g/kg ethanol (+) by oral gavage. (B) Hepatic mRNA expression of the indicated genes in WT mice 1 h after giving either water (–) or 1 g/kg ethanol (+) by oral gavage plus vehicle or p300/CBP inhibitor (C646, 20 mg/kg) by i.p. injection. Values are means ± SEM. *$p < 0.05$, **$p < 0.01$ and ****$p < 0.0001$ for water versus ethanol gavage groups by two-way ANOVA with Tukey's multiple comparisons test.

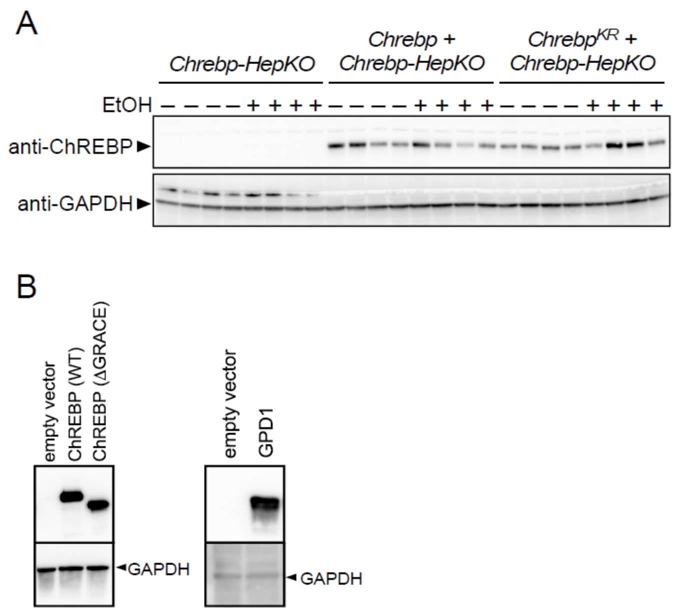

Fig. S2. ChREBP protein expression after reintroduction into *Chrebp* knockout livers.
(A) Hepatic expression of ChREBP proteins in *Chrebp-HepKO* mice in which either WT ChREBP or the acetylation sites mutant $ChREBP^{KR}$ were reintroduced by AAV infection. Mice were killed 1 h after giving water (–) or 1 g/kg ethanol (+) by oral gavage. (B) Expression of WT ChREBP, ChREBP-ΔGRACE and GPD1 proteins in transfected HEK-293T cells.

Table S1. List of QPCR primers.

Mouse gene	Forward primer	Reverse primer
Cyclophilin	GGAGATGGCACAGGAGGAA	GCCCGTAGTGCTTCAGCTT
Fgf21	CCTCTAGGTTTCTTTGCCAACAG	AAGCTGCAGGCCTCAGGAT
Chrebpβ	TCTGCAGATCGCGTGGAG	CTTGTCCCGGCATAGCAAC
G6pc	GTGGCAGTGGTCGGAGACT	ACGGGCGTTGTCCAAAC
Txnip	AGCCAGCCAACTCAAGAGGC	CAGCATGGATGGAGATGTCATC
Klf10	GGGTGTGGCAAGACTTACTTTAAA	GAGCGAGCAAACCTCCTTTC
Mlx	TGCAGAGAAGACAGCTCTCACC	CCTCATCATCTGTGTTGGGGAC
